# Special Hospitals—Lying in and Women

**Published:** 1892-10-08

**Authors:** 


### HOSPITAL ADMINISTRATION.

### IV.—SPECIAL HOSPITALS: LYING-IN AND WOMEN.

#### Cost of Management.*

It is not easy to deal with the lying-in hospitals. They occupy a place entirely different from general hospitals, owing to the character of the work done, and the nature of the cases. As a rule the patients reside but a very short time in the wards; their general health is good from first to last, and the expenditure upon the in-patients can therefore be reduced to a relatively small sum. The secretary of a lying-in hospital has, however, to raise the necessary income by the same methods as other hospital authorities, and so the cost of management to maintenance must be treated on the same lines. We are doubtful, however, if lying-in hospitals excite at the present day anything like equal sympathy with the general and children's hospitals, and bearing in mind the comparatively small size of these institutions, it would be reasonable to expect that the percentage of cost of administration to that of maintenance would average upwards of 15 per cent., though it should not exceed 20 per cent. Bearing this in mind, let us see how the figures work out.

#### Metropolitan Lying-In Hospitals.

| Name of Hospital. | Beds for Occupation. | Average Number Occupied. | Total In-Patients Admitted. | Total Out-Patients Admitted. | Percentage of Cost of Administration to that of Maintenance. |
|---|---|---|---|---|---|
| Queen Charlotte's | 58 | 37 | 942 | 1,148 | 11·445 |
| City of London | 36 | 22 | 455 | 1,555 | 8 724 |
| General Lying-In | 24 | 19 | 463 | 1,248 | 9·7 |
| Clapham Maternity | 20 | 9 | 162 | 267 | 9·885 |
| British Lying-In | 16 | 8 | 170 | 508 | 10·913 |
| East-End Mothers' Home | 12 | 4 | 86 | 28 | 43 800 |

Mr. Owthwaite is to be congratulated that, although he has charge of the second largest lying-in hospital, the cost of administration to that of maintenance is less than 9 per cent. This is a remarkable result, which reflects great credit upon the Committee and Secretary, and seems to point to the fact that the latter is, to say the least, not over-paid for the very excellent services which he renders to this charity. It will be seen that, with two exceptions, the percentage of cost of administration is remarkably small, a fact which reflects the greatest credit upon all concerned. The average cost of administration of all the hospitals proper is less than half the maximum sum—which we put down at 20 per cent.—which it is reasonable to allow for the expenses of management. We are informed that exceptional circumstances—not likely to recur—have produced the extraordinary result that the cost of administration at the East-end Mothers' Home has reached the enormous sum of 43·8 per cent., or about 9s. in the pound. This excessive outlay has prevented the Committee of Distribution of the Hospital Sunday Fund from making a grant to this useful institution, the work of which is excellent, and the value of which to the mothers of the East-end cannot be exaggerated.

* Previous articles of this series have appeared in The Hospital, page 365 for August 27th, page 381 for September 3rd, and page 13 for October 1st.

#### Diseases Peculiar to Women.

There are six hospitals in London which confine their attention to the diseases peculiar to women, but of these two—the Grosvenor and the Royal—admit a certain number of children also. This is a time when women's rights are much to the fore, and there never has been an age more in sympathy with the best interests of women than that in which we live. These considerations lead us to conclude that 15 per cent. is a fair average sum to lay down as the maximum which may be spent by this class of institution upon management. The following is a list of the metropolitan women's hospitals.

#### Metropolitan Hospitals for Women.

| Name of Hospital. | Beds for Occupation. | Average Number Occupied. | Total In-Patients Admitted. | Total Out-Patients Admitted. | Percentage of Cost of Administration to that of Maintenance. |
|---|---|---|---|---|---|
| Hospital for Women, Soho Square | 66 | 51 | 670 | 5,375 | 20·248 |
| Chelsea Hospital | 60 | 38 | 494 | 3.539 | 23·119 |
| Samaritan Free | 53 | 47 | 572 | 7,073 | 14·723 |
| Royal Hospital, Lambeth | 51 | 48 | 586 | 7,126 | 19·225 |
| New Hospital | 42 | 24 | 304 | 4,435 | 15·177 |
| Grosvenor | 18 | 13 | 108 | 1,715 | 11·627 |

The foregoing is a remarkable table in many ways. First, we have the anomalous position that the greater the number of beds in women's hospitals in London at the present time, the greater is the expenditure upon the management. Thus, the expenses of management of three of these institutions, viz., the Chelsea, the Hospital for Women, and the Royal Hospital for Children and Women is 5 per cent. higher than it should be, if our estimate is a reasonable one, as we believe it to be. We hope the authorities will give some explanation of the causes which have produced this extraordinary result, having regard to the working of the remaining three hospitals for women, and of those devoted to lying-in cases. The New Hospital for Women is entirely managed by women, and it is greatly to the credit of the managers, in view of the foregoing facts, that the expenses of management should be, in round numbers, only 15 per cent. The Grosvenor Hospital, a relatively small institution, with only 18 beds, spends less than 12 per cent. on management, a fact which makes us commend it to the liberal support of the charitable. The Samaritan Free Hospital, probably the best known of all women's hospitals, which is famous for the work done within its walls by Sir Spencer Wells, and which is under the management of one of the ablest of hospital secretaries, Mr. George Scudamore, spends less than 15 per cent. upon management. This circumstance confirms the justice of our contention that 15 per cent. is an adequate sum to allow for the management expenses of women's hospitals. Mr. Scudamore has had many and great difficulties to contend with, and it is not too much to say that the results brought out by these figures bear eloquent testimony not only to the efficiency of his work, but to the care which he exercises in regard to one of the best-managed of special hospitals. We believe that the Samaritan Free Hospital and the New Hospital for Women are, on the whole, the most efficiently-conducted of women's hospitals, and it is satisfactory to find that, despite the fact that they contain every modern appliance, and that the internal administration leaves little or nothing to be desired, the percentage of cost of administration to that of maintenance should be comparatively so small. We commend these facts to the attention of the Committee of the New Hospital and the Samaritan Free Hospital, and we hope they may see their way to increase the emoluments given to the chief officials, whose devoted labours have secured these eminently satisfactory results.

